# Crystal structure of the phosphorylated *Arabidopsis* MKK5 reveals activation mechanism of MAPK kinases: Activation mechanism of AtMKK5

**DOI:** 10.3724/abbs.2022089

**Published:** 2022-07-18

**Authors:** Chao-Jun Pei, Qing-Xia He, Zhipu Luo, Hongwei Yao, Zhi-Xin Wang, Jia-Wei Wu

**Affiliations:** 1 Institute of Molecular Enzymology School of Biology & Basic Medical Sciences Suzhou Medical College of Soochow University Suzhou 215123 China; 2 MOE Key Laboratory for Protein Science School of Life Sciences Tsinghua University Beijing 100084 China

**Keywords:** MAPK signaling, *Arabidopsis* MKKs, kinase interaction motif, active conformation of MKK, kinetic study

## Abstract

The mitogen-activated protein kinase (MAPK) signaling pathways are highly conserved in eukaryotes, regulating various cellular processes. The MAPK kinases (MKKs) are dual specificity kinases, serving as convergence and divergence points of the tripartite MAPK cascades. Here, we investigate the biochemical characteristics and three-dimensional structure of MKK5 in *Arabidopsis* (AtMKK5). The recombinant full-length AtMKK5 is phosphorylated and can activate its physiological substrate AtMPK6. There is a conserved kinase interacting motif (KIM) at the N-terminus of AtMKK5, indispensable for specific recognition of AtMPK6. The kinase domain of AtMKK5 adopts active conformation, of which the extended activation segment is stabilized by the phosphorylated Ser221 and Thr215 residues. In line with sequence divergence from other MKKs, the αD and αK helices are missing in AtMKK5, suggesting that the AtMKK5 may adopt distinct modes of upstream kinase/substrate binding. Our data shed lights on the molecular mechanisms of MKK activation and substrate recognition, which may help design specific inhibitors targeting human and plant MKKs.

## Introduction

Mitogen-activated protein kinase (MAPK) cascades are highly conserved signaling modules in eukaryotes ranging from yeast to mammals [1]. A conventional MAPK pathway contains three sequentially activated kinases, termed MAPK kinase kinase (MAP3K or MEKK), MAPK kinase (MAP2K, MKK or MEK) and MAPK. MAP3Ks, the topmost member of the tripartite cascades, are activated by signals received from receptors/sensors, and then phosphorylate MKKs on their serine and threonine residues in the S/T-X _3-5_-S/T motif. The phosphorylated MKKs can activate MAPKs through phosphorylating threonine and tyrosine residues in the activation segment [ 2, 3] . These kinases have been implicated in a wide array of biological processes including cell differentiation, proliferation, migration and apoptosis [ 4– 6] .

For mammals, there are four subfamilies of MAPKs, known as the extracellular signal-regulated kinases (ERK1/2), the c-Jun N-terminal kinases (JNK1/2/3), the p38α/β/γ/δ kinases and ERK5. Seven human MKKs have been identified to activate specific MAPKs: MEK1/2 for ERK1/2, MKK4/7 for JNKs, MKK3/6 for p38 kinases, and MEK5 for ERK5 [ 7, 8] . These dual-specificity MKKs at the middle of the three-tier MAPK cascades share common structural features to ensure the specificity of signal transmission. The N-terminal flexible region contains a kinase interaction motif (KIM) specifically binding to cognate upstream MAP3Ks and downstream MAPKs [ 9– 12] . The conserved kinase domains of human MKKs are 200~300 amino acids in length, responsible for the transfer reaction of ATP γ-phosphate [13]. As with most of other protein kinases, the catalytic activities of MKKs are directly related to the phosphorylation of key Ser/Thr residues within their activation segments, which might induce conformational changes [ 14– 16] . To understand the regulatory mechanism, the three-dimensional structures of MEK1/2 and MKK4/6/7 have been determined [ 17– 23] . However, all reported human MKK structures were of non-phosphorylated proteins adopting inactive conformation.

The MAPK cascades are evolutionarily conserved in plants, mediating the signaling of abiotic stresses, pathogens as well as plant hormones. Some 60 MAP3Ks, 10 MKKs and 20 MAPKs (also termed MPKs, for plant proteins) have been identified in *Arabidopsis*, which can combine into distinct MAPK cascades [ 24– 26] . The *Arabidopsis thaliana* MPKs (AtMPKs) can be classified into four subgroups (designated A–D) based on sequence similarities [2]. Groups A, B and C possess a TEY phosphorylation motif in their activation segments similar to animal ERK subfamily (thereby known as ERK-like AtMPKs), while the TDY signature for Group D AtMPKs is plant-specific. The AtMKKs are also divided into four subgroups: Group A includes AtMKK1/2 and AtMKK6; Group B only includes AtMKK3; Group C contains AtMKK4/5; and Group D comprises AtMKK7–10. *Arabidopsis* MAP3Ks are divergent in sequence and structure and poorly characterized in MAPK signaling pathways. Two best-characterized cascades (named by MKK-MPK core) are the AtMKK4/5-AtMPK3/6 and AtMKK1/2-AtMPK4 pathways [ 5, 6] . However, little is known about the molecular mechanisms for activity regulation and cognate recognition of these *Arabidopsis* kinases.

AtMKK5 has been shown to play roles in plant growth and development processes, including the embryogenesis, stomatal development, floral organ abscission, stress response, and defense response [ 27– 34] .

In this study, we showed biochemical evidence that the full-length AtMKK5 is phosphorylated and can catalyze the dual phosphorylation of AtMPK6. The KIM of AtMKK5 was shown to play an essential role in specific recognition and effective activation of AtMPK6 *in vitro*. We also solved the crystal structure of AtMKK5 kinase domain at 3.2 Å. Combined with our biochemical studies, we demonstrated that phosphorylation at both Thr215 and Ser221 residues within the activation segment is required to establish the active conformation of AtMKK5 and important for the kinase activity towards AtMPK6. AtMKK5 also shows unique structural features, which may account for the distinct substrate specificity from human MKKs.

## Materials and Methods

### Materials

ATP, phosphoenolpyruvate (PEP), NADH, lactate dehydrogenase (LDH), pyruvate kinase (PK), *p*-nitrophenyl phosphate ( *p*NPP) and 7-methyl-6-thioguanosine (MESG) were purchased from Sigma (Saint Louis, USA). 3-(N-morpholino) propane sulfonic acid (MOPS) was purchased from Amresco (Renton, USA). Other chemicals were local products of analytical grade (Sinopharm, Shanghai, China) used without further purification. Ultrapure water was used throughout.

### Protein preparation

The full-length AtMKK5 and various fragments were amplified by standard PCR and cloned into pET28b vector with N-terminal His _6_-tag. The AtMPK6 and lambda protein phosphatase (λPP) reconstructed in pGEX4T-2 vectors were expressed as the N-terminal GST-tagged proteins. The full-length AtMKK5 protein was coexpressed with λPP for dephosphorylation. All mutants of AtMKK5 and AtMPK6 were generated by overlap PCR and verified by DNA sequencing. All proteins were overexpressed in *Escherichia coli* BL21 (DE3) cells at 18°C with 0.2 mM IPTG induction. The subsequent protein purification was performed by using Ni-NTA (Qiagen, Hilden, Germany) or GS4B (GE Healthcare, Uppsala, Sweden) affinity columns, the Source-15Q anion exchange column and Superdex 200 10/300 GL gel filtration column on an AKTA FPLC (GE Healthcare, Uppsala, Sweden) at 4ºC, and the purity was verified by SDS-PAGE. The purified proteins were stored at –80ºC, and stocks directed at kinase activity assays were supplemented with glycerol to a final concentration of 20% (v/v). Protein concentrations were determined spectrophotometrically based on theoretical molar extinction coefficients at 280 nm [35].

### Crystallization and structure determination

All proteins were crystallized by vapor-diffusion technique in hanging drops, and the drops were prepared by mixing equal volumes of protein with reservoir solution at 18ºC. The crystal of kinase domain of AtMKK5 (AtMKK5-KD) was grown in a reservoir solution containing 0.1 M Bis-Tris propane (pH 7.0), 2.8 M NaAc (pH 7.0) and 0.4 M NaCl, cryo-protected in reservoir solutions supplemented with 10% glycerol, and then flash frozen in liquid nitrogen. The diffraction data were collected at beamline 17U at Shanghai Synchrotron Radiation Facility (Shanghai, China) and processed with the HKL2000 package [36]. The crystal belongs to space group *P*2 _1_2 _1_2 _1_ and each asymmetric unit comprises eight molecules. The structure was solved by molecular replacement using Phaser [37] with human MKK6 (PDB: 3VN9) as the search model, and refined to 3.2 Å resolution with a *R*_work_ / *R*_free_ of 0.211/0.240 using programs REFMAC5 [38] and Coot [39]. Data collection and refinement statistics are shown in Table 1. The atomic coordinates and structure factors have been deposited in the Protein Data Bank with accession code 7XBR. All structural representations in this paper were prepared with PyMOL ( http://www.pymol.org).

**Table:** **Table 1** Statistics of data collection and refinement

	AtMKK5-KD
**Data collection ^a^**	
Space group	*P*2 _1_2 _1_2 _1_
Cell dimensions	
*a*, *b*, *c* (Å)	83.7, 198.6, 202.8
α, β, γ (º)	90, 90, 90
Resolution (Å)	50.00–3.20 (3.31–3.20) ^b^
Unique reflections	55113 (5452)
*R*_merge_	0.167 (1.697)
*R*_pim_	0.063 (0.625)
*I* /σ ( *I)*	15.6 (1.9)
Completeness (%)	96.7 (97.2)
Redundancy	7.5 (7.6)
*CC*_1/2_	0.990 (0.872)
	
**Refinement**	
Resolution (Å)	49.13–3.20 (3.28–3.20)
No. reflections	52111 (3814)
*R*_work_ / *R*_free_	0.211 / 0.240
No. atoms	
Protein	16189
Ligand/ion	392
*B*-factors	
Protein	108.3
Ligand/ion	147.8
R.m.s. deviations	
Bond lengths (Å)	0.009
Bond angles (º)	1.670
Ramachandran plot	
Favored regions (%)	95.79
Allowed regions (%)	4.21
Outliers regions (%)	0

^a^The data set was collected from a single crystal.

^b^Values in parentheses are for the highest-resolution shell.

### Kinetic analyses of AtMKK5

The ATPase activity of AtMKK5 was determined using a spectrophotometric assay coupling the production of ADP with the oxidation of NADH by PK and LDH [40]. This assay was carried out at 25°C in a 1.8 mL reaction mixture containing the assay buffer (50 mM MOPS, pH 7.0, 100 mM NaCl, 0.1 mM EDTA, 10 mM MgCl _2_, 0.2 mM NADH, 1.0 mM PEP, 15 units/mL PK, and 20 units/mL LDH) and varying amounts of ATP. The reactions were initiated by the addition of AtMKK5 to the reaction mixture. Progress of the reaction was monitored continuously by following NADH oxidation at 340 nm on the Lambda 45 spectrophotometer equipped with a magnetic stirrer in the cuvette holder (PerkinElmer, Waltham, USA), and the concentration of ADP generated in the AtMKK5-catalyzed ATP hydrolysis reaction was determined using an extinction coefficient for NADH of 6220 cm ^–1^ M ^–1^ at 340 nm. The initial rates were determined from the linear slopes of the progress curves at indicated ATP concentrations, and the kinetic parameters ( *k*_cat_ and *K*_m_) were obtained by fitting the experimental data to the Michaelis-Menten equation using a nonlinear regression analysis program.

The kinase activity of AtMKK5 was measured spectrophotometrically using AtMPK6(K92M) as substrate. This kinase assay also couples ADP production to NADH oxidation, and the absorbance change at 340 nm was recorded continuously. The reaction was initiated by adding AtMKK5 to a reaction mixture containing the assay buffer, 1 mM ATP and different concentrations of protein substrate. The concentrations of phosphorylated AtMPK6 generated in the AtMKK5-catalyzed reactions were determined using the extinction coefficient for NADH of 6220 cm ^–1^ M ^–1^ at 340 nm, and the experimental data were then analysed using the nonlinear regression program.

### Dephosphorylation of AtMKK5-KD

The dephosphorylation of phosphorylated AtMKK5-KD was determined using a continuous spectrophotometric assay [41]. The experiment was performed at 25°C in 1.8 mL of standard assay buffer containing 50 mM MOPS (pH 7.0), 100 mM MESG, and 0.1 mg/mL *p*NPP. The reactions were initiated by the addition of λPP, and the continuous absorbance changes at 360 nm were recorded. The change in absorbance was due to the conversion of MESG to 7-methyl-6-thioguanine in the presence of inorganic phosphate released from the dephosphorylation of the phosphorylated AtMKK5-KD by phosphatase. The quantity of released phosphate was determined using an extinction coefficient of 11,200 cm ^–1^ M ^–1^ for the phosphate-dependent reaction at 360 nm [42].

### Immunoblotting

The phosphorylation states of AtMKK5 and AtMPK6 were analyzed by western blot analysis using specific antibodies. Protein samples were resolved by SDS-PAGE and then electro-transferred onto polyvinylidene difluoride (PVDF) membranes (Merck Millipore, Darmstadt, Germany). After incubation of PVDF membranes with anti-phospho-Thr, anti-phospho-Tyr, anti-bisphosphorylated ERK1/2 (Cell Signaling Technology, Danvers, USA; cat. no. 9381, 9411, and 4370), and anti-phospho-Ser antibodies (Abcam, Cambridge, UK; cat. no. ab9332), specific immuno-complexes were detected by chemiluminescence using ECL reagents (Beyotime, Shanghai, China). The PVDF membrane was finally exposed to X-ray film (Kodak, Rochester, USA).

### Gel filtration assays

The apparent molecular weight of AtMKK5 in solution and the interaction between AtMKK5 and AtMPK6 were assessed at 4ºC by size-exclusion chromatography using Superdex 200 10/300 column (GE Healthcare). All protein samples were incubated at 4°C for 60 min to reach equilibrium. The column was equilibrated with the buffer containing 10 mM Tris-HCl (pH 8.0), 150 mM NaCl, and 2 mM dithiothreitol (Amresco, Renton, USA) at a flow rate of 0.5 mL/min and calibrated with molecular mass standards. All fractions were collected at 0.5 mL each, and aliquots of relevant fractions were subjected to SDS-PAGE followed by Coomassie blue staining.

## Results and Discussion

### Biochemical characterization of the recombinant AtMKK5

**Figure 1:**
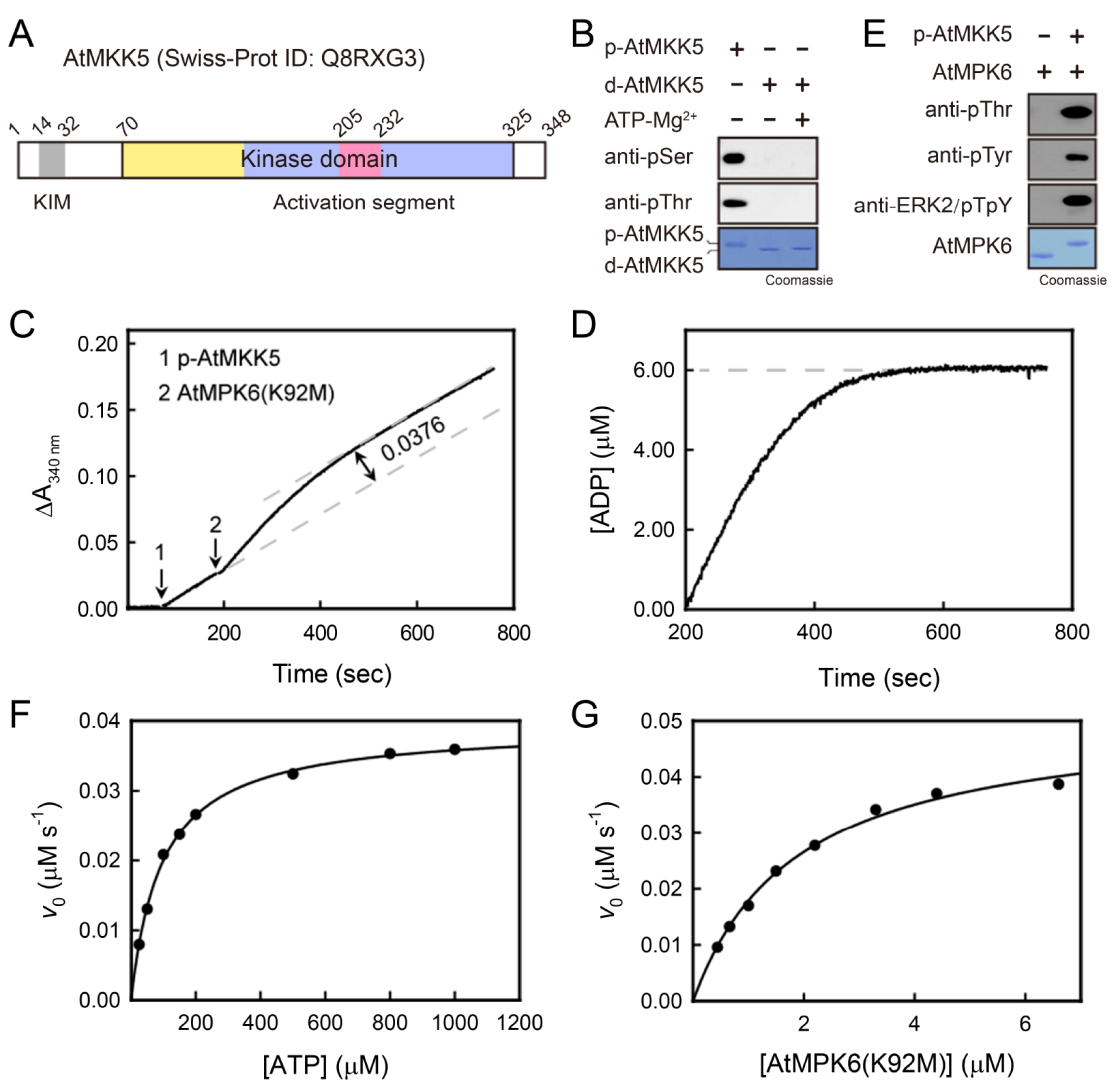
Figure 1 AtMKK5 effectively phosphorylates AtMPK6 at both Thr and Tyr sites (A) Schematic diagram of AtMKK5. The structural elements are colored as follows: KIM (grey), kinase domain (N-lobe, yellow; C-lobe, light blue; activation segment, pink). (B) Phosphorylation states of AtMKK5 analyzed using anti-pSer and anti-pThr antibodies. (C) Time courses of AtMKK5-catalyzed ATP hydrolysis and AtMPK6 phosphorylation. The additions of p-AtMKK5 (20 nM) and AtMPK6(K92M) (3.0 μM) to the reaction mixture (containing 1 mM ATP) are indicated. (D) Time course of AtMPK6 phosphorylation after subtracting AtMKK5-catalyzed ATP hydrolysis. (E) Phosphorylation states of AtMPK6 analyzed using anti-pThr, anti-pTyr and anti-ERK2/pTpY antibodies. (F–G) Dependence of the initial rate of AtMKK5-catalyzed reaction on the concentration of ATP (F) and AtMPK6(K92M) (G). The reaction mixture contained 20 nM p-AtMKK5 in standard assay buffer, and K m for AtMPK6 was measured at 1 mM ATP. The solid lines are the best fitting result according to the Michaelis-Menten equation.

The full-length AtMKK5 contains a conventional kinase domain (residues 70–325) flanked by two flexible regions ( Figure 1A). We purified the full-length AtMKK5 over-expressed in *E*. *coli*, and found that the recombinant protein was phosphorylated at threonine and serine residues ( Figure 1B). A notable property of certain protein kinases is their ability to undergo autophosphorylation and autoactivation [ 43, 44] . The phosphorylated AtMKK5 (p-AtMKK5) can be dephosphorylated by the generic protein phosphatase λPP, producing the fully dephosphorylated AtMKK5 (d-AtMKK5). However, d-AtMKK5 did not undergo autophosphorylation in the presence of 1 mM ATP and 10 mM Mg ^2+^ *in vitro* ( Figure 1B). The recombinant full-length AtMKK5 might be phosphorylated by *E*. *coli* kinases or autophosphorylated because of the high concentration during expression.

Upon phosphorylation of their activation segments, many protein kinases, such as human p38 and MEK1, possess basal hydrolytic activity toward ATP [ 45, 46] . The ATPase activity of p-AtMKK5 was monitored continuously by a spectrophotometric assay coupling ADP production to NADH oxidation ( Figure 1C). Addition of p-AtMKK5 to the reaction mixture resulted in a fast production of ADP, and the steady-state rate determined by the slope of the reaction profile was a measure of p-AtMKK5 ATPase activity. In *Arabidopsis*, AtMPK6 has been reported to be the physiological substrate downstream of AtMKK5 [27]. To eliminate the effect of ATP hydrolysis catalyzed by the phosphorylated AtMPK6, an inactive mutant AtMPK6(K92M) was used as the protein substrate. Further addition of AtMPK6(K92M) into the reaction mixture induced a burst (ΔA _340_=0.0376), followed by the steady production of ADP ( Figure 1C). The initial burst was attributed to the additional production of ADP due to the phosphorylation of AtMPK6 by AtMKK5, and the steady-state rate again reflected the ATPase activity of p-AtMKK5. The progress curve after subtracting the AtMKK5-catalyzed ATP hydrolysis represents AtMPK6 phosphorylation by AtMKK5 alone, which was near 100% complete in 300 s ( Figure 1D). Quantitation of ADP production was determined using the extinction coefficient of 6220 cm ^–1^ M ^–1^ for NADH oxidation at 340 nm, which clearly indicated that approximate 6 μM ADP was generated upon the complete phosphorylation of 3.0 μM AtMPK6(K92M). Thus, the phosphorylation stoichiometry was determined to be close to 2 mol of ADP/mol of AtMPK6. Western blot analysis using specific anti-ERK2/pTpY antibody corroborated that the recombinant p-AtMKK5 can phosphorylate both Thr221 and Tyr223 in the conserved TEY motif of AtMPK6 ( Figure 1E) [ 27, 28] .

We then determined the kinetic parameters for the ATP hydrolyzing and protein phosphorylating activities of p-AtMKK5, respectively. In the absence of protein substrate, a typical set of initial velocities versus ATP concentration is shown in Figure 1F, and curve fitting of the data to the Michaelis-Menten equation yielded the *k*_cat(ATP)_ and *K*_m(ATP)_ values of 1.96 ± 0.02 s ^–1^ and 96.2 ± 3.5 μM, respectively. As to the p-AtMKK5-catalyzed AtMPK6(K92M) phosphorylation, the initial velocity, after substracting the ATPase activity, also gave rise to the hyperbolic dependence on the protein concentration, with a *k*_cat(AtMPK6)_ of 2.57± 0.09 s ^–1^ and *K*_m(AtMPK6)_ of 1.88± 0.15 μM ( Figure 1G). The substrate specificity constant *k*_cat_/ *K*_m_ for p-AtMKK5-catalyzed AtMPK6 phosphorylation was 1.37×10 ^6^ M ^–1^ s ^–1^, comparable to those of the dual-phosphorylated human MEK1 and phospho-mimicking mutant MKK6EE with respect to their cognate MAPKs (2.73×10 ^6^ M ^–1^ s ^–1^ and 1.20×10 ^6^ M ^–1^ s ^–1^) [ 47, 48] . Therefore, AtMKK5 is the effective upstream kinase of AtMPK6.

### The N-terminal KIM of AtMKK5 is indispensable for AtMPK6 recognition

**Figure 2:**
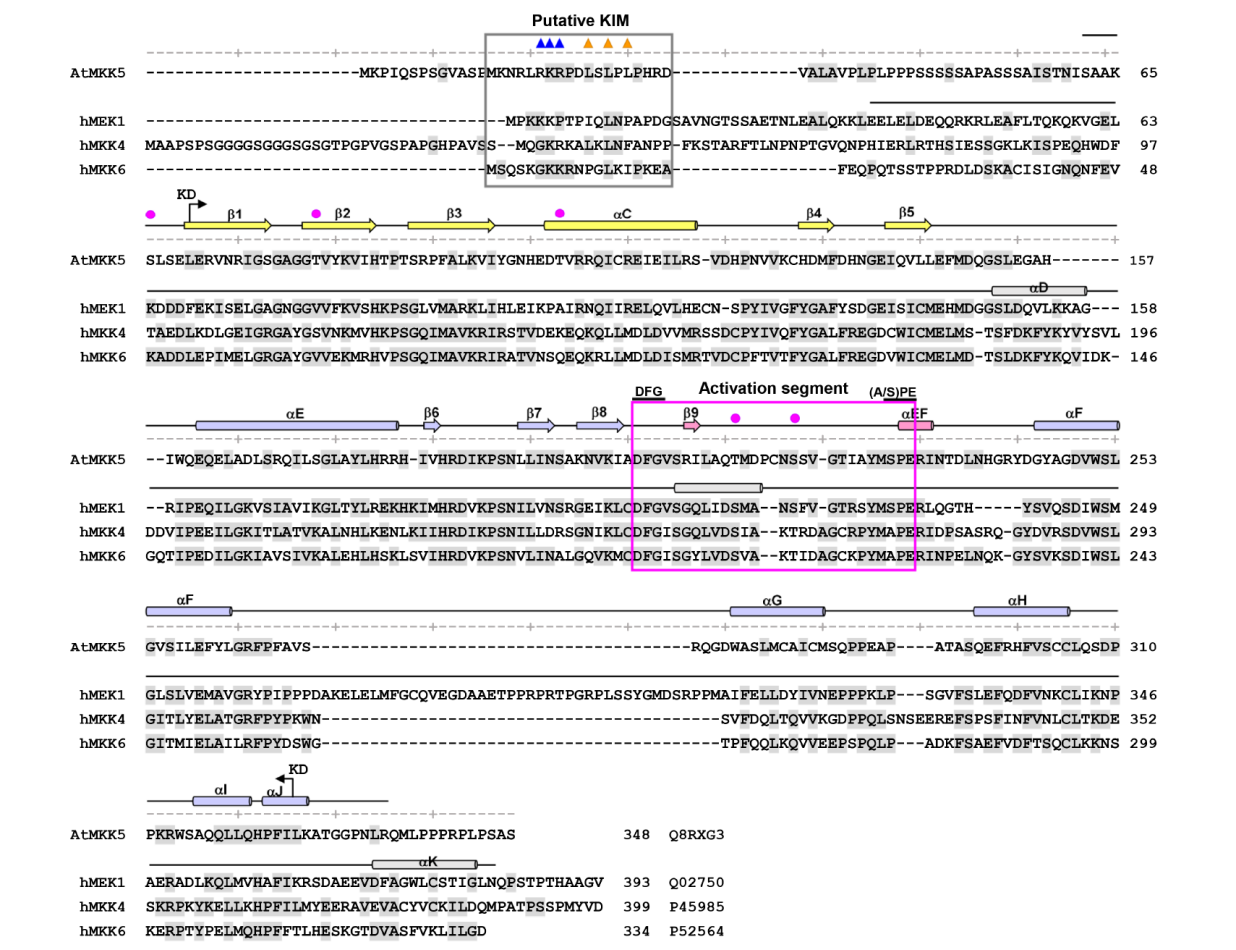
Figure 2 Sequence alignment of AtMKK5 with hMKKs The putative KIM and the activation segment are boxed, and the conserved DFG and (A/S)PE motifs at two termini of the activation segment are indicated. Only the distinct secondary structures in the hMEK1 are shown. Residues in the KIM sequences involved in substrate recognition are indicated by triangles (charged, blue; hydrophobic orange), and the phosphorylation sites within the activation segment and in the N-lobe are highlighted by magenta circles. The code at the end of each MKK sequence is the corresponding Swiss-Prot ID.

**Figure 3:**
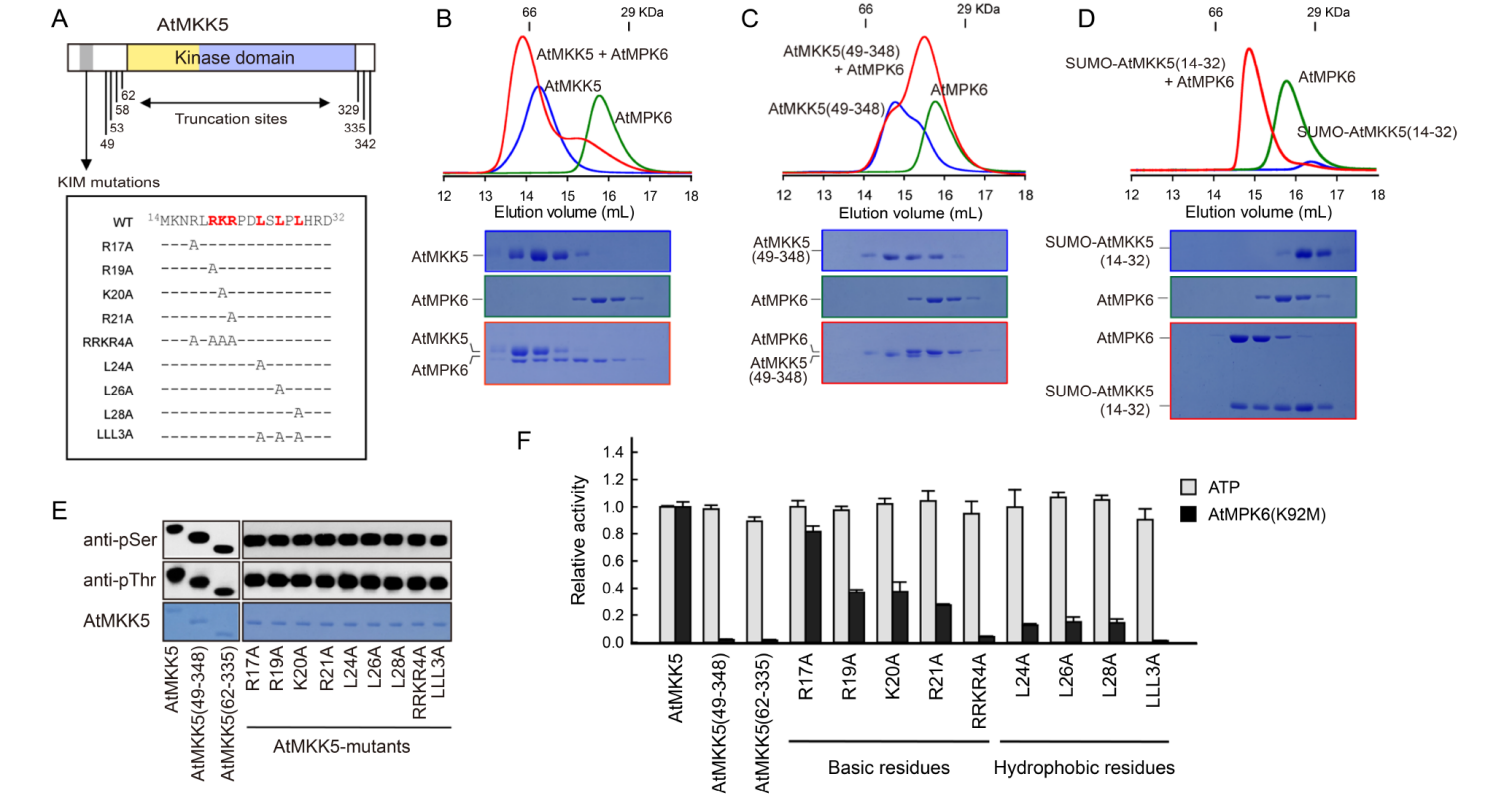
Figure 3 The conserved KIM of AtMKK5 is indispensable for AtMPK6 phosphorylation (A) Schematic illustration of AtMKK5 truncation sites and KIM mutations. The KIM residues involved in AtMPK6 recognition are highlighted in red and bold. (B–D) Recognition of AtMPK6 by the full-length AtMKK5 (B), the representative N-terminal truncation AtMKK5(49–348) (C) and the KIM peptide alone (D) analyzed using size exclusion chromatography. (E) Phosphorylation states of AtMKK5 mutants detected by western blot analysis. (F) Comparison of the ATP-hydrolyzing and AtMPK6-phosphorylation activities of wild-type and mutant AtMKK5 (mean±SEM, n=3). The reaction mixture includes 20 nM AtMKK5 and 1 mM ATP, without or with 1 μM AtMPK6(K92M).

All mammalian MKKs possess the conserved KIM sequence in their N-terminal noncatalytic regions, which are required for their efficient recognition, phosphorylation of cognate MAPKs [49]. A putative KIM sequence (residues 14–32) was found in the N-terminal extension of AtMKK5 ( Figure 2) [ 2, 50– 52] . To assess the function of this segment, we generated a number of N-terminal truncation variants and evaluated their abilities to interact with AtMPK6 using gel filtration assays ( Figure 3A). When equal amounts of AtMKK5 and AtMPK6 were incubated and analyzed, AtMPK6 co-migrated with the full-length AtMKK5 to earlier fractions ( Figure 3B). In the absence of the putative KIM, the AtMKK5(49–348) mutant was barely able to form complex with AtMPK6 ( Figure 3C). Moreover, the putative KIM peptide with N-terminal His _6_-SUMO was subcloned and purified, and the gel filtration analysis clearly demonstrated that the KIM alone was able to interact with AtMPK6 ( Figure 3D). However, the C-terminal deletion mutations of AtMKK5 had little, if any, effect on AtMPK6 recognition. These data suggested that the N-terminal region, most likely the putative KIM, plays an essential role in the interaction between AtMKK5 and AtMPK6.

The consensus sequence of KIM is comprised of two major elements, an N-terminal cluster of basic residues and a C-terminal hydrophobic motif of the form Φ _A_-X-Φ _B_ [11]. To demonstrate the importance of the KIM sequence, we generated a series of point mutations on the full-length AtMKK5 ( Figure 3A). These site-specific mutants, as well as two N-terminal truncations, exhibited similar phosphorylation signals of Ser/Thr residues to the wild-type AtMKK5 ( Figure 3E). We then evaluated the effects of these mutations on the ATPase and kinase activities of AtMKK5 ( Figure 3F). All mutants displayed comparable ATP hydrolyzing activities; however, the catalytic activities of most mutants towards AtMPK6 were greatly reduced. The AtMKK5(49–348) and AtMKK5(62–335) mutants without the predicted KIM sequence were unable to carry out substrate phosphorylation. When the four basic residues (Arg17, Arg19, Lys20 and Arg21) in the KIM sequence of AtMKK5 were individually replaced by Ala, the catalytic activities of three mutants (R19A, K20A and R21A) were decreased by about 3-folds, while the R17A mutation showed the modest effect ( Figure 3F). When three Leu residues in the C-terminal hydrophobic portion of AtMKK5 KIM were individually replaced by Ala, the catalytic activities of mutants L24A, L26A and L28A for AtMPK6 phosphorylation were decreased approximately by 10 folds. Compared to wild-type AtMKK5, substituting all four Arg/Lys residues with Ala simultaneously (RRKR4A) resulted in a significant decrease (by ~25 folds), and replacement of all three hydrophobic residues (LLL3A) led to a 90-fold drop in the initial velocity. These data clearly demonstrated that the KIM sequence of AtMKK5, both the basic cluster and the hydrophobic Φ _A_-X-Φ _B_ motif, is indispensable for the phosphorylation and activation of AtMPK6, while the hydrophobic motif appears to be the major contributor in the specific recognition of AtMPK6 by AtMKK5.

### Overall structure of AtMKK5 kinase domain

**Figure 4:**
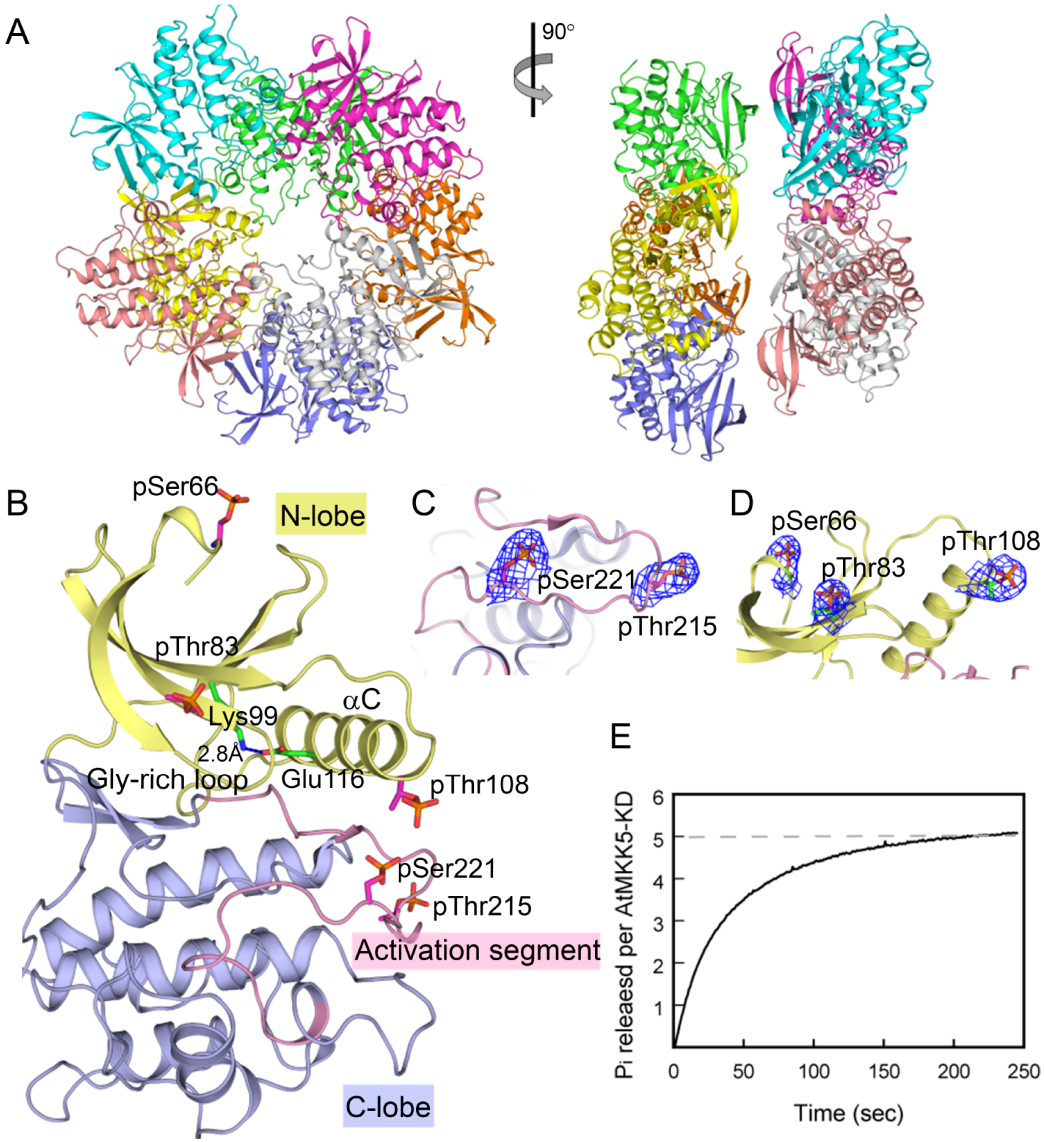
Figure 4 Crystal structure of the phosphorylated AtMKK5-KD (A) Schematic representations of eight AtMKK5 molecules within one asymmetric unit in two views. (B) Representative overall structure of AtMKK5-KD (molecule C). The color scheme followed that in Figure1A. Some catalytically critical elements and the distance between conserved Lys and Glu side chains are indicated, and the phosphorylated residues are highlighted as magenta sticks. (C,D) The 2 Fo-Fc omit maps (contoured at 1.2 σ) for two conserved phosphorylation sites within the activation segment (C) and three additional residues in the N-lobe (D). (E) Time course for the dephosphorylation of recombinant AtMKK5-KD. The reaction mixture contains 1 μM AtMKK5-KD and 200 nM λPP.

We next carried out crystallization trials on the full-length and truncation proteins. One truncation mutant containing just the kinase domain (residues 62–335, hereafter referred to as AtMKK5-KD) yielded crystals, diffracted to a resolution of 3.2 Å ( Table 1). The structure was determined by molecular replacement, and each asymmetric unit contains eight molecules forming a double-layered ring ( Figure 4A). The eight molecules adopt essentially the same conformation, except that the Gly-rich loop between strands β1 and β2 for nucleotide binding is visible only for molecule C. The structural analyses hereafter were performed on the basis of molecule C ( Figure 4B). The overall structure of AtMKK5-KD exhibits the classical bilobal kinase fold, comprising a small N-lobe (residues 64–149) and a large C-lobe (150–329). The N-lobe contains an antiparallel β-sheet and the essential αC helix, and the C-lobe includes six α-helices and two catalytically critical elements (the catalytic loop and the activation segment).

Interestingly, five phosphorylated Ser/Thr residues were well resolved on the electron density map of AtMKK5-KD, two highly conserved phosphorylation sites within the activation segment (pThr215 and pSer221) and three additional residues in the N-lobe (pSer66, pThr83 and pThr108) ( Figure 4C,D). Dephosphorylation of AtMKK5 kinase domain by λPP was monitored continuously by another coupled enzyme system, where the release of inorganic phosphate was detected using purine nucleoside phosphorylase and its chromophoric substrate MESG [41]. The phosphorylation stoichiometry was determined to be close to 5 mol of phosphate per mol of AtMKK5-KD ( Figure 4E). Thus, the structural and biochemical data clearly demonstrated that the recombinant AtMKK5-KD is phosphorylated at five Ser/Thr sites, including the two primary phosphorylation sites in the activation segment.

**Figure 5:**
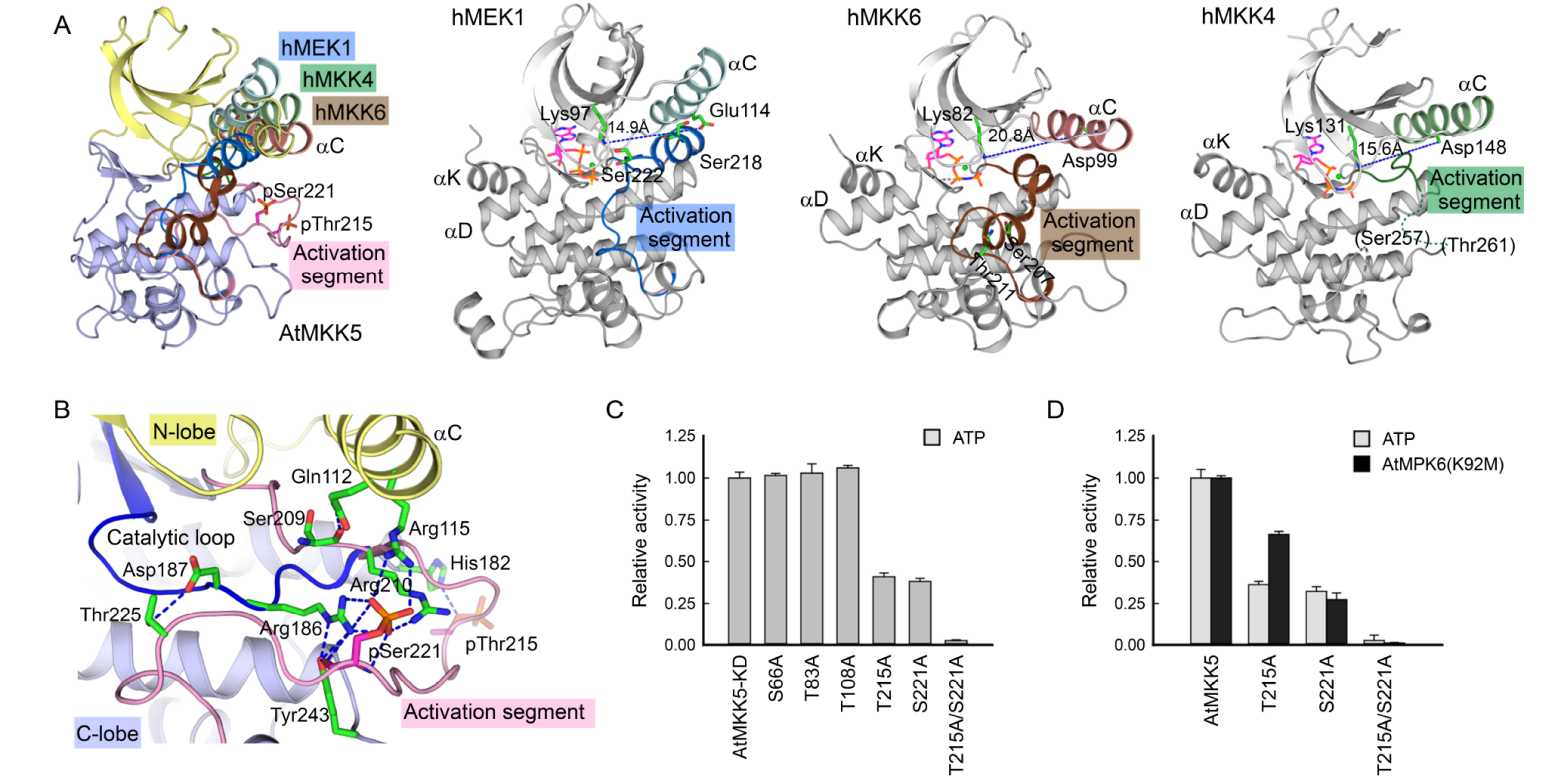
Figure 5 Phosphorylation of the activation segment is required for AtMKK5 activity (A) Comparison of the kinase domains from human MKKs with AtMKK5-KD. All structures are superimposed over their C-lobes, and AtMKK5 is colored as that in Figure4B. For clarity, only the helix αC and the activation segment of human MKKs are displayed and colored as follows: hMEK1 (palecyan, marine); hMKK6 (salmon, brown) and hMKK4 (palegreen, forest). In addition, the kinase domains of human MKKs are individually shown in grey, with the ATP analogues coloured in magenta. The phosphorylation sites and the K-E distances are indicated. (B) Close-up view of the phosphorylated activation segment. The catalytic loop is colored in blue, and residues interacting with pThr215 and pSer221 are highlighted as green sticks. (C) Effects of mutating the phosphorylation sites on the ATPase activity of AtMKK5-KD. The reaction mixture contains 20 nM AtMKK5-KD and 1 mM ATP (mean±SEM, n=3). (D) Comparison of the catalytic activities of full-length AtMKK5 bearing Thr215 and/or Ser221 mutations. The assays were performed with 20 nM AtMKK5 and 1 mM ATP, in the absence or presence of 1 μM AtMPK6(K92M).

Protein kinases are molecular switches which can adopt at least two conformations, active and inactive. This AtMKK5 structure is the first phosphorylated conformation determined for the MKK family, since all reported structures of human MKKs were crystallized using non-phosphorylated proteins [ 17– 23] . Many of human MKK structures are in complex with various inhibitors or determined using the phosphomimetic proteins. Thus, we compared the phosphorylated AtMKK5-KD structure only with the wild-type kinase domains of human MEK1, MKK6 and MKK4 in complex with ATP analogues (PDB IDs: 3EQD, 3VN9, and 3ALN). When superimposed on their kinase C-lobes, substantial conformational differences were revealed, in particular the orientation of the prominent αC helix and the conformation of the activation segment ( Figure 5A). The phosphorylated AtMKK5-KD adopts an active conformation similar to the prototype kinase, the catalytic subunit of cAMP-dependent protein kinase (PKA) [53]. By contrast, the non-phosphorylated human MKKs all display the inactive, αC-out conformation.

### The phosphorylated activation segment in AtMKK5-KD

The active kinases have several signature characteristics, such as the extended conformation of the activation segment and the proper orientation of helix αC [ 13, 14, 54, 55] . In our phosphorylated AtMKK5-KD structure, the activation segment of AtMKK5 between and including the DFG and (A/S)PE motifs (residues 205–232) adopts an extended conformation, protruding out from the catalytic cleft between the N- and C-lobes ( Figure 4B). Notably, the phosphate moiety of pSer221 fits snugly into a positively charged pocket formed by three guanidine groups of Arg115 from the αC helix, Arg186 of the catalytic HRD motif, and Arg210 within the activation segment ( Figure 5B). The pThr215 residue, by contrast, is largely exposed to solvent, only interacting with the imidazolium moiety of His182 from helix αE in the C-lobe. Moreover, several main-chain carbonyl and amide groups from the activation segment, including that of pSer221, form multiple hydrogen bonds with residues from helix αC in the N-lobe and from the catalytic loop and the αEF-αF loop in the C-lobe. Together, these interactions stabilize the extended conformation of the activation segment and the relative orientation of two lobes. In particular, the phosphorylated Ser221 in AtMKK5 takes the same position as pThr197 in the active PKA conformation, crucial for the proper orientation of helix αC and the correct electrostatic environment for the catalytic base (Asp187 of AtMKK5 and Asp166 of PKA) ( Figure 5B). Consequently, the prominent αC helix in the phosphorylated AtMKK5 adopts the typically active conformation, where the important Glu116 on helix αC interacts with Lys99 from strand β3 ( Figure 4B).

Similar to most other inactive protein kinases, the activation segment of the unphosphorylated human MKK4 is partially invisible in the electron density map; however, the activation segments of inactive MEK1 and MKK6 are traceable and folded back into the catalytic cleft ( Figure 5A). Interestingly, their N-terminal portions display α-helical conformation, distinct from that in the phosphorylated, active AtMKK5 ( Figure 2). Three residues of the MEK1 activation segment form multiple hydrogen bonds with residues from helix αC (including the important Glu114), and the N-terminal short helix within the MKK6 activation segment packs against the αC helix mainly via hydrophobic contacts. The folded activation segments in MEK1 and MKK6 lead to the inactive, αC-out conformations, where the critical acidic residues (Glu114 of MEK1, and Asp99 in MKK6) are 15~20 Å away from the important Lys on strand β3 ( Figure 5A). Remarkably, the key Ser/Thr side chains in the phosphorylation motifs of human MKKs are in close contacts with their neighboring residues, and phosphorylation of these Ser/Thr residues would disrupt the inactive, fold-in conformations of their activation segments. Therefore, the phosphorylated AtMKK5 structure provides insights into the phosphorylation-induced conformational changes of the activation segment, as well as the inward movement of helix αC.

In addition to pThr215 and pSer221 within the activation segment, three phosphorylated residues were assigned in the N-lobe of AtMKK5 kinase domain. The phosphate groups of pSer66 at the N-terminal extension and pThr108 on the αC helix are hydrogen bonded to their neighboring residues respectively, while the side chain of pThr83 on strand β2 makes no interaction. To assess the importance of the five phosphorylation sites, we individually substituted these Ser/Thr residues with Ala and examined their effects on the catalytic function of AtMKK5-KD ( Figure 5C). Replacement of Thr215 and Ser221 in the activation segment led to approximately 3-fold reduction in the ATPase activity, while substitution of three amino acids in the N-lobe had little, if any, effect on the basal activity. Remarkably, when Thr215 and Ser221 were simultaneously replaced by Ala, the ATP-hydrolyzing ability of AtMKK5-KD was severely affected. We also mutated the key Thr215 and Ser221 in the background of full-length AtMKK5, and their effects on the ATPase activity were the same as those AtMKK5-KD mutants ( Figure 5D). The kinase activities of two single mutants T215A and S221A were decreased by 2~3 folds, and the double mutant T215A/S221A only retained about 3% catalytic activity towards AtMPK6. These data clearly demonstrated that two primary phosphorylation sites Thr215 and Ser221 in the activation segment are both required for the catalytic activity of AtMKK5.

### The ATP and protein substrate binding sites in AtMKK5

**Figure 6:**
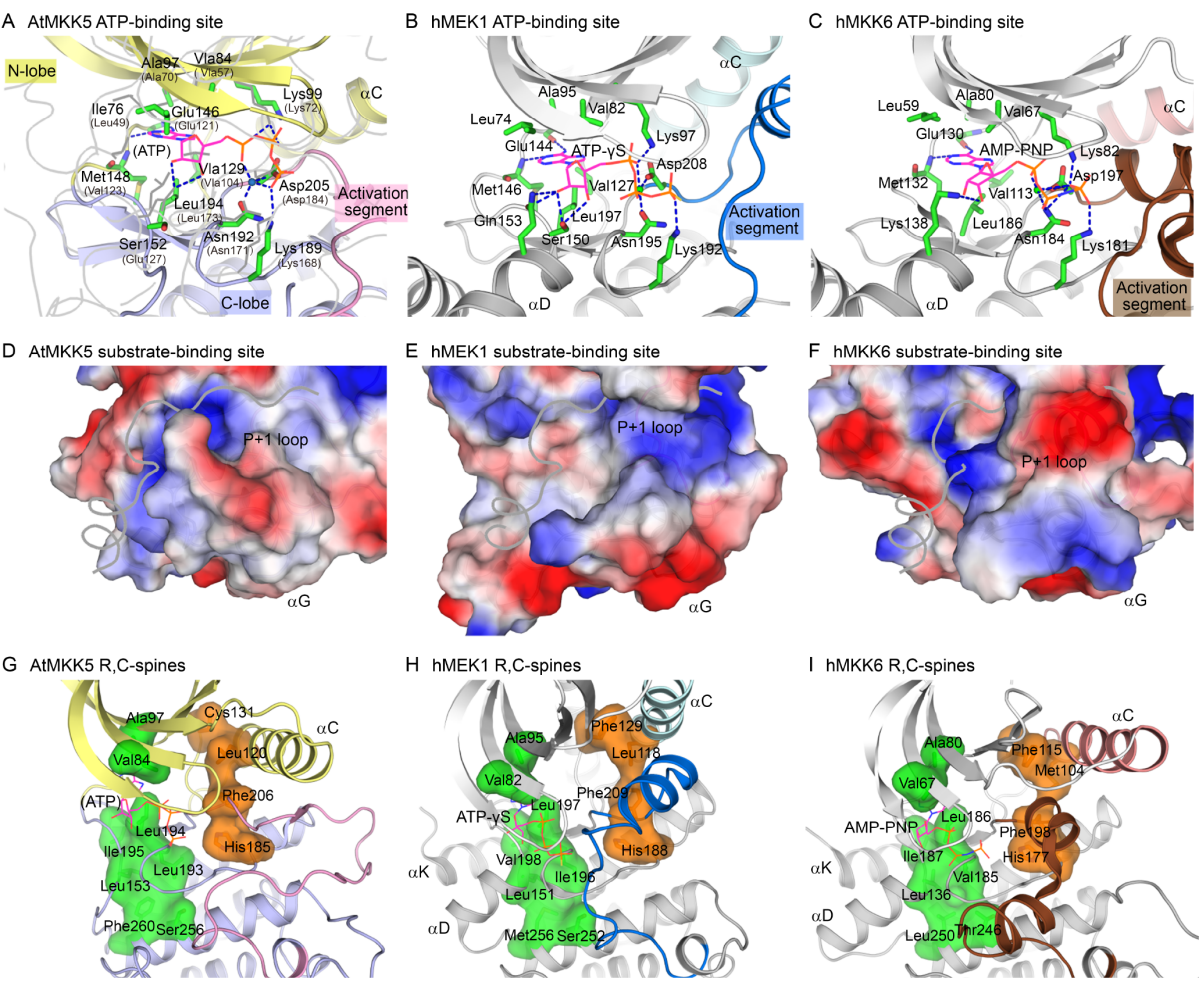
Figure 6 Comparison of the active site conformations of phosphorylated AtMKK5 and unphosphorylated hMKKs (A–C) Close-up views of the ATP-binding sites in AtMKK5, hMEK1 and hMKK6. In panel A, the ATP molecule adapted from PKA (ATP) is shown as magenta lines, and the ATP-interacting residues of PKA are shown as grey lines and labeled in parentheses. The corresponding residues of AtMKK5 are highlighted as green sticks. (D–F) Close-up views of the protein substrate-binding sites in AtMKK5, hMEK1 and hMKK6. The kinase domains are shown in surface representation, colored according to the electrostatic potential (positive, blue; negative, red). The peptide substrate adapted from the PKA structure is colored grey. (G–I) Comparison of the C- and R-spines in AtMKK5, hMEK1 and hMKK6. Residues in the C-spine and the R-spine are highlighted as green and orange sticks, respectively, and additionally in surface model.

Protein kinases catalyze the transfer of the γ-phosphate of ATP to Ser/Thr/Tyr residues of protein substrate. We tried to co-crystallize AtMKK5 with ATP and/or AtMPK6, but all efforts failed. An ATP molecule was adapted from the active PKA structures, since the ATP analogues bind in different conformations to the non-phosphorylated, inactive human MKKs ( Figure 6A–C). The adenine ring of ATPγS analogue nestles in the hydrophobic pocket formed mainly by residues from the N-lobe β-sheet of PKA (and AtMKK5), while the ribose moiety and triphosphate group form multiple hydrogen bonds with charged or polar side chains mainly from the C-lobe ( Figure 6A). The hinge region connecting the kinase N- and C-lobes further stabilizes ATP binding via main-chain hydrogen bonds and van der Waals interactions with the adenine ring. In addition, the DFG motif at the N-terminus of the activation segment contributes to the correct positioning of ATP phosphate groups in the active PKA, and this conserved motif in our AtMKK5 structure displays similar DFG-in configuration ( Figure 6A). In particular, the carboxyl group of invariant Asp205 is correctly oriented to chelate the ATP-bound magnesium ion, and Phe206 makes hydrophobic contacts with residues on helix αC. Therefore, almost all the ATP-interacting residues in the AtMKK5-KD structure are well positioned, clearly demonstrating that the ATP binding site of the phosphorylated AtMKK5 is largely configured in the absence of ATP•Mg ^2+^.

The P+1 loop, located at the C-terminus of the activated segment, is a critical point for the binding of protein substrate [56]. This loop in the AtMKK5 structure (residues 222–231/232) adopts an extended conformation, capable of accommodating a protein/peptide substrate as that in the PKA-peptide complex structure ( Figure 6D). By contrast, the P+1 loops in the inactive MKK structures are folded into the cleft, which would hinder the binding of an exogenous substrate ( Figure 6E,F). In the proximity of the P+1 site, a basic groove is observed in AtMKK5, whereas the corresponding region in human MEK1 is hydrophobic and flat ( Figure 6D,E). The positively-charged characteristics of this groove in AtMKK5 are consistent with the presence of negatively charged residues around the phosphorylation sites of its substrate AtMPK6. Notably, helix αG in many protein kinases is also involved in substrate recognition. The αG helix of AtMKK5 is mostly hydrophobic, while those in human MKKs are largely charged ( Figure 6D–F). These structural differences between plant and human MKKs might determine their substrate specificities, as well as their distinct physiological functions in variant signaling pathways.

We also investigated two functionally important “spines”, the catalytic spine (C-spine) and the regulatory spine (R-spine), both of which are anchored to helix αF that is deeply buried in the C-lobe ( Figure 6G–I). The C-spine consisting of eight conserved residues is optimally arranged, where three hydrophobic residues (Aal84, Ala97 and Leu194 of AtMKK5) from both N- and C-lobes contribute to the accommodation of ATP adenine ring ( Figure 6G). The R-spine comprises four nonconsecutive residues from the catalytically important elements on both lobes (the αC helix, the DFG motif and the catalytic loop), and these highly conserved residues in the phosphorylated AtMKK5 join the N- and C-lobes through considerable hydrophobic interactions. The C-spines of human MKKs are appropriately configured; however, the R-spines (or helix αC) adopt distorted conformations for inactive kinases ( Figure 6H,I). The proper assembly of both R- and C-spines further corroborates that the phosphorylated AtMKK5 adopts an active conformation.

### Sequence and structural differences between plant and human MKKs

**Figure 7:**
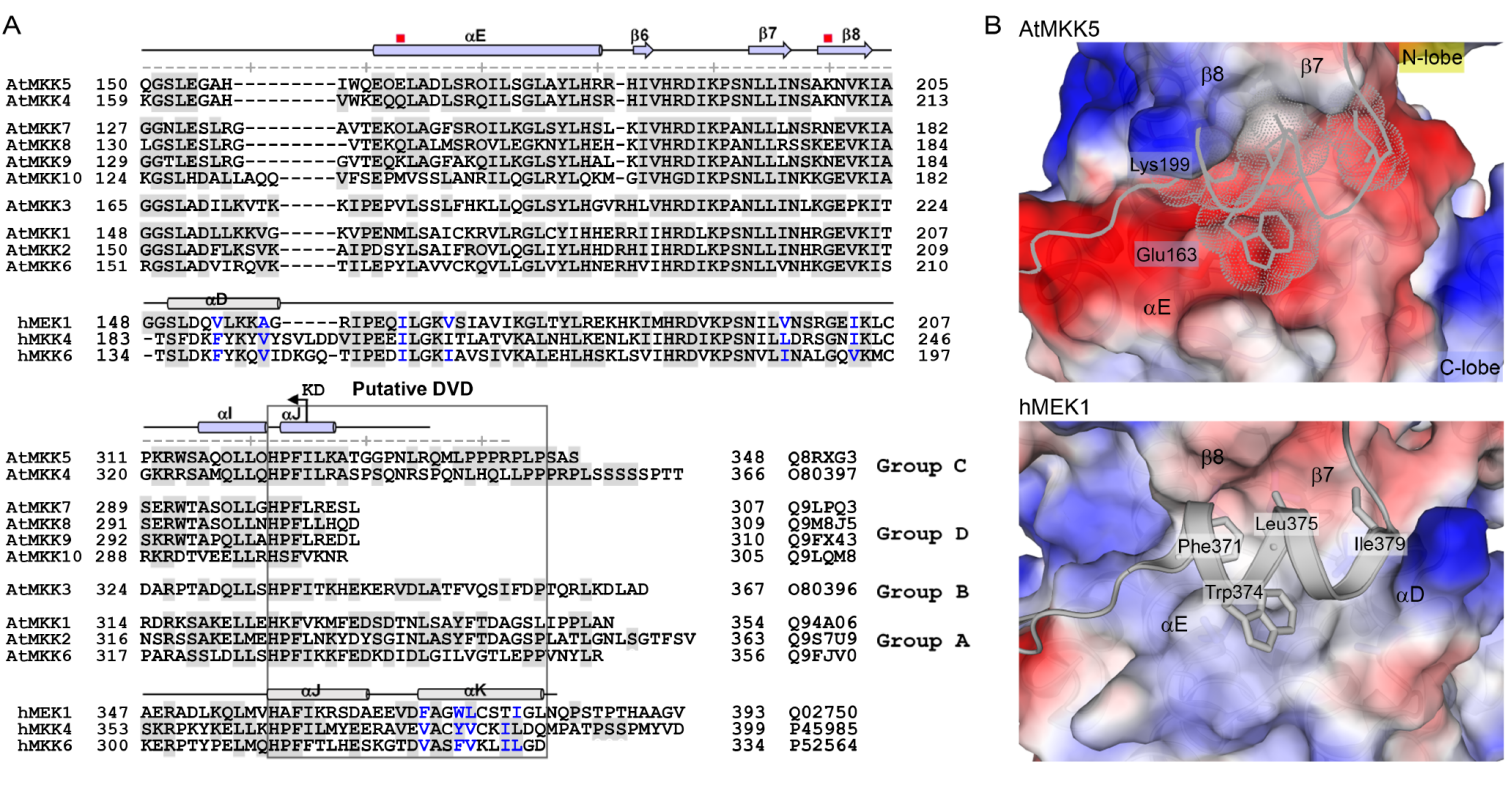
Figure 7 Structural differences between plant and human MKKs (A) Sequence alignment of the kinase C-lobes from Arabidopsis and human MKKs. The αD-helix regions and putative DVD sites are boxed. The αK-interacting residues in human MKKs are highlighted in blue, and two key non-conserved residues in AtMKK5 are indicated by red squares. (B) Comparison of the DVD sites in AtMKK5 and hMEK1. The kinase domains, except helices αK, are shown in surface representation, and the αK helix adapted from hMEK1 (grey ribbon) is superimposed onto AtMKK5.

Interestingly, one ATP-interacting residue is located differently in plant and human MKKs ( Figure 6A-C). A charged or polar residue located on helix αD in the C-lobe of human MKKs can coordinate the ribose moiety of ATP; however, this αD helix is absent in the pAtMKK5-KD structure, replaced by a stretched loop (residues 152–157). Sequence analyses revealed that the corresponding region in AtMKK5 is shortened when compared with those in human MKKs, and that the loss of helix αD may also occur in some other *Arabidopsis* MKKs ( Figures 2 and 7A). Notably, the αD helix in human MKKs not only accommodates the ATP molecule, but also interacts with helix αK ( Figure 5A). In human MKKs, helix αK binds in a hydrophobic groove formed by helices αD, αE and strands β7, β8; however, the corresponding groove on AtMKK5 is somehow charged ( Figure 7B). Two charged side chains of Glu163 from helix αE and Lys199 on strand β8 would preclude the binding of helix αK in AtMKK5, and such charged/polar and bulky amino acids are also found in four other *Arabidopsis* MKKs ( Figure 7A).

The C-terminal αK- and αJ-helices in human MKKs were reported to constitute a docking site termed DVD (domain for versatile docking), and mutations of some hydrophobic residues in this site could reduce the interaction of MKKs with upstream MAP3Ks [57]. The corresponding regions of AtMKK4/5 are not conserved to human MKKs, and the Group D *Arabidopsis* MKKs even lack the sequence for helix αK ( Figure 7A). In line with the sequence differences, the DVD site observed in human MKKs is missing in AtMKK5 (and most likely AtMKK4, members of Group D). By contrast, sequence analysis implied that AtMKKs of Groups A and B might possess a DVD site. Thus, it would be interesting to unravel the different mechanisms by which *Arabidopsis* MKKs recognize their cognate MAP3Ks.

## Conclusions

The MKKs are central kinases in various tripartite MAPK cascades. We found that the recombinant AtMKK5 protein is phosphorylated at Thr and Ser residues, exhibiting both ATPase and kinase activities. The kinetic studies demonstrated that AtMKK5 is an effective upstream kinase of AtMPK6, phosphorylating both Thr221 and Tyr223 in the TXY motif with a substrate specificity constant *k*_cat_/ *K*_m_ of 1.37×10 ^6^ M ^–1^ s ^–1^. The conserved KIM in the N-terminal extension of AtMKK5 is indispensable for the specific recognition and activation of AtMPK6, and mutagenesis analysis indicated that the hydrophobic Φ _A_–X–Φ _B_ motif is the major determinant. To elucidate the molecular mechanism, we determined the crystal structure of AtMKK5-KD to a resolution of 3.2 Å, where two highly conserved phosphorylated residues (pThr215 and pSer221) were well resolved in the electron density map. To our knowledge, this is the first phosphorylated, active MKK structure. Structural comparison with the non-phosphorylated, inactive MKK structures revealed substantial conformational changes of the activation segment and the prominent αC helix. The phosphorylated Ser221 not only stabilizes the extended conformation of the activation segment, but also interacts with residues from helix αC and the catalytic loop to modulate their proper orientation and correct electrostatic environment. By contrast, pThr215 only interacts with one side chain from the C-lobe. Nevertheless, our mutation data demonstrated that both Thr215 and Ser221 are required for the physiological function of AtMKK5. Detailed structural analyses showed that the ATP-binding site in the phosphorylated AtMKK5-KD structure is largely configurated through in the absence of ATP•Mg ^2+^. The protein substrate binding site, including the P+1 loop, is well-formed and positively charged, capable of accommodating the negatively charged side chain(s) in the proximity of AtMPK6 phosphorylation sites. Interestingly, consistent with the sequence divergency, helices αD and αK observed in human MKK structures are replaced by a loop or even missing in AtMKK5, yet might present in certain plant MKKs. Further structure and function studies may provide insights into the distinct mechanisms by which plant MKKs guarantee the specific MAPK-MKK-MAP3K cascades.

## References

[1] Widmann C, Gibson S, Jarpe MB, Johnson GL (1999). Mitogen-activated protein kinase: conservation of a three-kinase module from yeast to human. Physiol Rev.

[2] Ichimura K, Shinozaki K, Tena G, Sheen J, Henry Y, Champion A (2002). Mitogen-activated protein kinase cascades in plants: a new nomenclature. Trends Plant Sci.

[3] Roberts PJ, Der CJ (2007). Targeting the Raf-MEK-ERK mitogen-activated protein kinase cascade for the treatment of cancer. Oncogene.

[4] Kyriakis JM, Avruch J (2012). Mammalian MAPK signal transduction pathways activated by stress and inflammation: a 10-year update. Physiol Rev.

[5] Meng X, Zhang S (2013). MAPK cascades in plant disease resistance signaling. Annu Rev Phytopathol.

[6] Xu J, Zhang S (2015). Mitogen-activated protein kinase cascades in signaling plant growth and development. Trends Plant Sci.

[7] Cargnello M, Roux PP (2011). Activation and function of the MAPKs and their substrates, the MAPK-activated protein kinases. Microbiol Mol Biol Rev.

[8] Akinleye A, Furqan M, Mukhi N, Ravella P, Liu D (2013). MEK and the inhibitors: from bench to bedside. J Hematol Oncol.

[9] Weston CR, Lambright DG, Davis RJ (2002). MAP kinase signaling specificity. Science.

[10] Garai Á, Zeke A, Gógl G, Törő I, Fördős F, Blankenburg H, Bárkai T (2012). Specificity of linear motifs that bind to a common mitogen-activated protein kinase docking groove. Sci Signal.

[11] Peti W, Page R (2013). Molecular basis of MAP kinase regulation. Protein Sci.

[12] Zhou T, Raman M, Gao Y, Earnest S, Chen Z, Machius M, Cobb MH (2004). Crystal structure of the TAO2 kinase domain. Structure.

[13] Endicott JA, Noble MEM, Johnson LN (2012). The structural basis for control of eukaryotic protein kinases. Annu Rev Biochem.

[14] Johnson LN, Noble MEM, Owen DJ (1996). Active and inactive protein kinases: structural basis for regulation. Cell.

[15] Roskoski Jr. R (2019). Targeting ERK1/2 protein-serine/threonine kinases in human cancers. Pharmacol Res.

[16] Annunziata MC, Parisi M, Esposito G, Fabbrocini G, Ammendola R, Cattaneo F (2020). Phosphorylation sites in protein kinases and phosphatases regulated by formyl peptide receptor 2 signaling. Int J Mol Sci.

[17] Ohren JF, Chen H, Pavlovsky A, Whitehead C, Zhang E, Kuffa P, Yan C (2004). Structures of human MAP kinase kinase 1 (MEK1) and MEK2 describe novel noncompetitive kinase inhibition. Nat Struct Mol Biol.

[18] Fischmann TO, Smith CK, Mayhood TW, Myers Jr. JE, Reichert P, Mannarino A, Carr D (2009). Crystal structures of MEK1 binary and ternary complexes with nucleotides and inhibitors. Biochemistry.

[19] Matsumoto T, Kinoshita T, Kirii Y, Yokota K, Hamada K, Tada T (2010). Crystal structures of MKK4 kinase domain reveal that substrate peptide binds to an allosteric site and induces an auto-inhibition state. Biochem Biophysl Res Commun.

[20] Matsumoto T, Kinoshita T, Matsuzaka H, Nakai R, Kirii Y, Yokota K, Tada T (2012). Crystal structure of non-phosphorylated MAP2K6 in a putative auto-inhibition state. J Biochem.

[21] Min X, Akella R, He H, Humphreys JM, Tsutakawa SE, Lee SJ, Tainer JA (2009). The structure of the MAP2K MEK6 reveals an autoinhibitory dimer. Structure.

[22] Sogabe Y, Hashimoto T, Matsumoto T, Kirii Y, Sawa M, Kinoshita T (2016). A crucial role of Cys218 in configuring an unprecedented auto-inhibition form of MAP2K7. Biochem Biophys Res Commun.

[23] Sogabe Y, Matsumoto T, Hashimoto T, Kirii Y, Sawa M, Kinoshita T (2015). 5Z-7-Oxozeaenol covalently binds to MAP2K7 at Cys218 in an unprecedented manner. BioOrg Medicinal Chem Lett.

[24] Yadav K, Jha SK, Sharma M, Pandey GK. Mitogen activated protein kinase: a versatile signaling cascade in plants.
J Proteins Proteom 2018, 9: 57–72. https://www.researchgate.net/publication/323377151_Mitogen_Activated_Protein_Kinase_A_Versatile_Signaling_Cascade_in_Plants.

[25] Chardin C, Schenk ST, Hirt H, Colcombet J, Krapp A (2017). Review: mitogen-activated protein kinases in nutritional signaling in arabidopsis. Plant Sci.

[26] Komis G, Šamajová O, Ovečka M, Šamaj J (2018). Cell and developmental biology of plant mitogen-activated protein kinases. Annu Rev Plant Biol.

[27] Asai T, Tena G, Plotnikova J, Willmann MR, Chiu WL, Gomez-Gomez L, Boller T (2002). MAP kinase signalling cascade in
*Arabidopsis* innate immunity. Nature.

[28] Wang H, Ngwenyama N, Liu Y, Walker JC, Zhang S (2007). Stomatal development and patterning are regulated by environmentally responsive mitogen-activated protein kinases in
*arabidopsis*. Plant Cell.

[29] Liu H, Wang Y, Xu J, Su T, Liu G, Ren D (2008). Ethylene signaling is required for the acceleration of cell death induced by the activation of AtMEK5 in
*Arabidopsis*. Cell Res.

[30] Meng X, Wang H, He Y, Liu Y, Walker JC, Torii KU, Zhang S (2012). A MAPK cascade downstream of ERECTA receptor-like protein kinase regulates
*arabidopsis* inflorescence architecture by promoting localized cell proliferation. Plant Cell.

[31] Xing Y, Chen W, Jia W, Zhang J (2015). Mitogen-activated protein kinase kinase 5 (MKK5)-mediated signalling cascade regulates expression of iron superoxide dismutase gene in
*Arabidopsis* under salinity stress. J Exp Bot.

[32] Zhang M, Wu H, Su J, Wang H, Zhu Q, Liu Y, Xu J (2017). Maternal control of embryogenesis by MPK6 and its upstream MKK4/MKK5 in
*Arabidopsis*. Plant J.

[33] Sun T, Nitta Y, Zhang Q, Wu D, Tian H, Lee JS, Zhang Y (2018). Antagonistic interactions between two
^MAP^ kinase cascades in plant development and immune signaling. EMBO Rep.

[34] Zhu Q, Shao Y, Ge S, Zhang M, Zhang T, Hu X, Liu Y (2019). A MAPK cascade downstream of IDA–HAE/HSL2 ligand–receptor pair in lateral root emergence. Nat Plants.

[35] Gill SC, von Hippel PH (1989). Calculation of protein extinction coefficients from amino acid sequence data. Anal Biochem.

[36] Otwinowski Z, Minor W. Processing of X-ray diffraction data collected in oscillation mode.
Methods Enzymol 1997, 276: 307–326. https://pubmed.ncbi.nlm.nih.gov/27754618/.

[37] McCoy AJ, Grosse-Kunstleve RW, Adams PD, Winn MD, Storoni LC, Read RJ (2007). *Phaser* crystallographic software. J Appl Crystlogr.

[38] Murshudov GN, Skubák P, Lebedev AA, Pannu NS, Steiner RA, Nicholls RA, Winn MD (2011). REFMAC5 for the refinement of macromolecular crystal structures. Acta Crystallogr D Biol Crystallogr.

[39] Emsley P, Cowtan K (2004). *Coot* : model-building tools for molecular graphics. Acta Crystlogr D Biol Crystlogr.

[40] Cook PF, Neville Jr. ME, Vrana KE, Hartl FT, Roskoski Jr. R (1982). Adenosine cyclic 3′,5′-monophosphate dependent protein kinase: kinetic mechanism for the bovine skeletal muscle catalytic subunit. Biochemistry.

[41] Killilea SD, Cheng Q, Wang ZX. Protein phosphatase type 1 and type 2A assays.
Methods Mol Biol 1998, 93: 23–33. https://doi.org/10.1385/0-89603-468-2:23.

[42] Sergienko EA, Srivastava DK (1994). A continuous spectrophotometric method for the determination of glycogen phosphorylase-catalyzed reaction in the direction of glycogen synthesis. Anal Biochem.

[43] Oh MH, Ray WK, Huber SC, Asara JM, Gage DA, Clouse SD (2000). Recombinant brassinosteroid insensitive 1 receptor-like kinase autophosphorylates on serine and threonine residues and phosphorylates a conserved peptide motif
*in vitro*. Plant Physiol.

[44] Wang J, Jiang J, Wang J, Chen L, Fan SL, Wu JW, Wang X (2014). Structural insights into the negative regulation of BRI1 signaling by BRI1-interacting protein BKI1. Cell Res.

[45] Chen G, Porter MD, Bristol JR, Fitzgibbon MJ, Pazhanisamy S (2000). Kinetic mechanism of the p38-α MAP kinase:  phosphoryl transfer to synthetic peptides. Biochemistry.

[46] Rominger CM, Schaber MD, Yang J, Gontarek RR, Weaver KL, Broderick T, Carter L (2007). An intrinsic ATPase activity of phospho-MEK-1 uncoupled from downstream ERK phosphorylation. Arch Biochem Biophys.

[47] Mansour SJ, Candia JM, Matsuura JE, Manning MC, Ahn NG (1996). Interdependent Domains controlling the enzymatic activity of mitogen-activated protein kinase kinase 1. Biochemistry.

[48] Wang YL, Zhang YY, Lu C, Zhang W, Deng H, Wu JW, Wang J (2019). Kinetic and mechanistic studies of p38α
^MAP^ kinase phosphorylation by
^MKK^ 6. FEBS J.

[49] Enslen H, Davis RJ (2001). Regulation of MAP kinases by docking domains. Biol Cell.

[50] Jiang M, Chu Z (2018). Comparative analysis of plant MKK gene family reveals novel expansion mechanism of the members and sheds new light on functional conservation. BMC Genomics.

[51] Tanoue T, Nishida E (2003). Molecular recognitions in the MAP kinase cascades. Cell Signalling.

[52] Bardwell L, Shah K (2006). Analysis of mitogen-activated protein kinase activation and interactions with regulators and substrates. Methods.

[53] Zheng J, Trafny EA, Knighton DR, Xuong NH, Taylor SS, Ten Eyck LF, Sowadski JM (1993). 2.2 Å refined crystal structure of the catalytic subunit of cAMP-dependent protein kinase complexed with MnATP and a peptide inhibitor. Acta Crystlogr D Biol Crystlogr.

[54] Taylor SS, Kornev AP (2011). Protein kinases: evolution of dynamic regulatory proteins. Trends Biochem Sci.

[55] Taylor SS, Shaw AS, Kannan N, Kornev AP (2015). Integration of signaling in the kinome: Architecture and regulation of the αC Helix. Biochim Biophys Acta (BBA) - Proteins Proteomics.

[56] Nolen B, Taylor S, Ghosh G (2004). Regulation of protein kinases. Mol Cell.

[57] Takekawa M, Tatebayashi K, Saito H (2005). Conserved docking site is essential for activation of mammalian MAP kinase kinases by specific MAP kinase kinase kinases. Mol Cell.

